# Impact of childhood traumatic brain injury on educational attainment in Finland from 1998 to 2018: a retrospective register-based nationwide cohort study

**DOI:** 10.1007/s10654-025-01218-9

**Published:** 2025-03-23

**Authors:** Julius Möttönen, Ilari Kuitunen, Ville T. Ponkilainen, Ville M. Mattila

**Affiliations:** 1 Faculty of Medicine and Life Sciences, University of Tampere, Kalevantie 3, 33100 Tampere, Finland; 2 Institute of Clinical Medicine, University of Eastern Finland, Kuopio, Finland; 3 Department of Pediatrics, Kuopio University Hospital, Kuopio, Finland; 4 Department of Orthopedics and Traumatology, Tampere University Hospital, Tampere, Finland

**Keywords:** Traumatic brain injury, Education, Cognition, Impairment

## Abstract

Pediatric traumatic brain injury (pTBI) can lead to considerable mortality, morbidity, mental impairment, and physical disability over time. The direct impact of pTBI on educational attainment is unclear. We included all pediatric 0- to 17-year-old patients who were at least 26 years old at the end of the follow-up with a diagnosis of TBI in the Finnish Care Registry for Health Care (years 1998 to 2018) to form our study group (pTBI group). The reference group comprised patients with ankle and wrist fractures. The pTBI group was further divided into concussions and specific intracranial injuries. We compared this information to Statistics Finland´s Degree/Qualification data to evaluate educational attainment at 3 main levels. All comparisons were made using logistic regression with 95% confidence intervals (CI). The pTBI group comprised 8 487 patients and the reference group comprised 15,552 patients. In total, 7594 pTBI patients had a concussion and 892 a specific intracranial injury. The pTBI group had lower odds of attaining any tertiary education compared with the reference group (odds ratio [OR] 0.85; CI 0.80, 0.90). The pTBI group was also more likely to remain at a lower tertiary education than attain higher tertiary education (OR 0.81; CI 0.74, 0.87). Patients with specific intracranial injuries were more likely not to attain any tertiary education compared to patients with concussions (OR 0.78; CI 0.68, 0.90). People with pTBI had lower educational attainment at all higher educational levels than the reference population with ankle and wrist injuries.

## Introduction

Traumatic brain injury (TBI) is defined as brain damage or disruption of brain function caused by a sudden external force [1]. Pediatric traumatic brain injuries (pTBIs) are common, with up to 280 per 100 000 annual emergency department diagnoses on a global scale [2]. TBIs are generally divided into 3 categories based on clinical findings and imaging results: mild, intermediate, and severe [1, 3, 4]. According to the most recent data, the incidence of mild pTBI has increased from 1998 through 2018 in Finland [5]. Despite the increasing incidence of mild pTBIs in Finland, the incidence of severe operatively treated pTBIs has remained the same [6].

Many studies show that pTBI can lead to considerable mortality, morbidity, mental impairment, and physical disability over time [7–15]. Pediatric TBIs are associated with long-lasting memory dysfunction and a wide range of adverse educational outcomes, such as the need for more supportive education, absenteeism, and exclusion [16–18]. Only a few studies have assessed the direct impact of pTBI on educational attainment. A nationwide Swedish cohort study suggested that people with childhood/adolescence TBI did not attain secondary education to the level of the general population [19]. The impact of TBI on education was already evident for mild brain injuries and increased according to the severity and higher age at the time of the initial injury [19]. However, the study lacked any detailed information on the educational levels and comparisons between different types of secondary education [19]. In an Australian questionnaire study with a small sample size, people with pTBI reported lower educational attainment [20]. A recent nationwide study in Wales found that especially injuries that were due to intentional self-harm, cyclist and pedestrian as well as high falls had negative impact on educational attainment [21]. There were also slight negative impact of minor TBI to the attainment, but more severe TBIs were not compared or investigated [21]. Lower socioeconomic status has shown to lead poorer recovery from TBI as well as negative correlation for educational attainment [21, 22].

Here, we assessed the impact of pTBI on educational attainment after secondary education compared with an orthopedically injured reference population in Finland. Our goal was to add more detailed and profound data on the impact of pTBI to educational attainment at all higher levels of education.

## Methods

We conducted a retrospective register-based nationwide cohort study in Finland. The data were assembled using 2 nationwide registers: the Finnish Care Register for Health Care and Statistic Finland´s Degree/Qualification-data. The study period lasted from January 1998 to December 2018 [23, 24].

### Finnish care register for health care

We included all 0- to 17-year-old pTBI patients from the Finnish Care Register for Health Care. The Register includes nationwide information on all hospital-level health care from secondary and tertiary levels in Finland. The patient group (pTBI group) included all emergency department visits and hospitalizations with an ICD-10 diagnostic code of S06 intracranial injury. We further used all subcodes of S06 to divide TBIs into concussions (S06.0) and more specific intracranial injuries on the basis of imaging findings (S06.1-S06.9). The reference group was designed to mimic similar risk-taking behavior as those patients with TBI and consisted of children with wrist or ankle fractures. A detailed list of all diagnosis codes and explanations is provided in Appendix 1.

### Statistic Finland´s degree/qualification-data

**Fig. 1:**
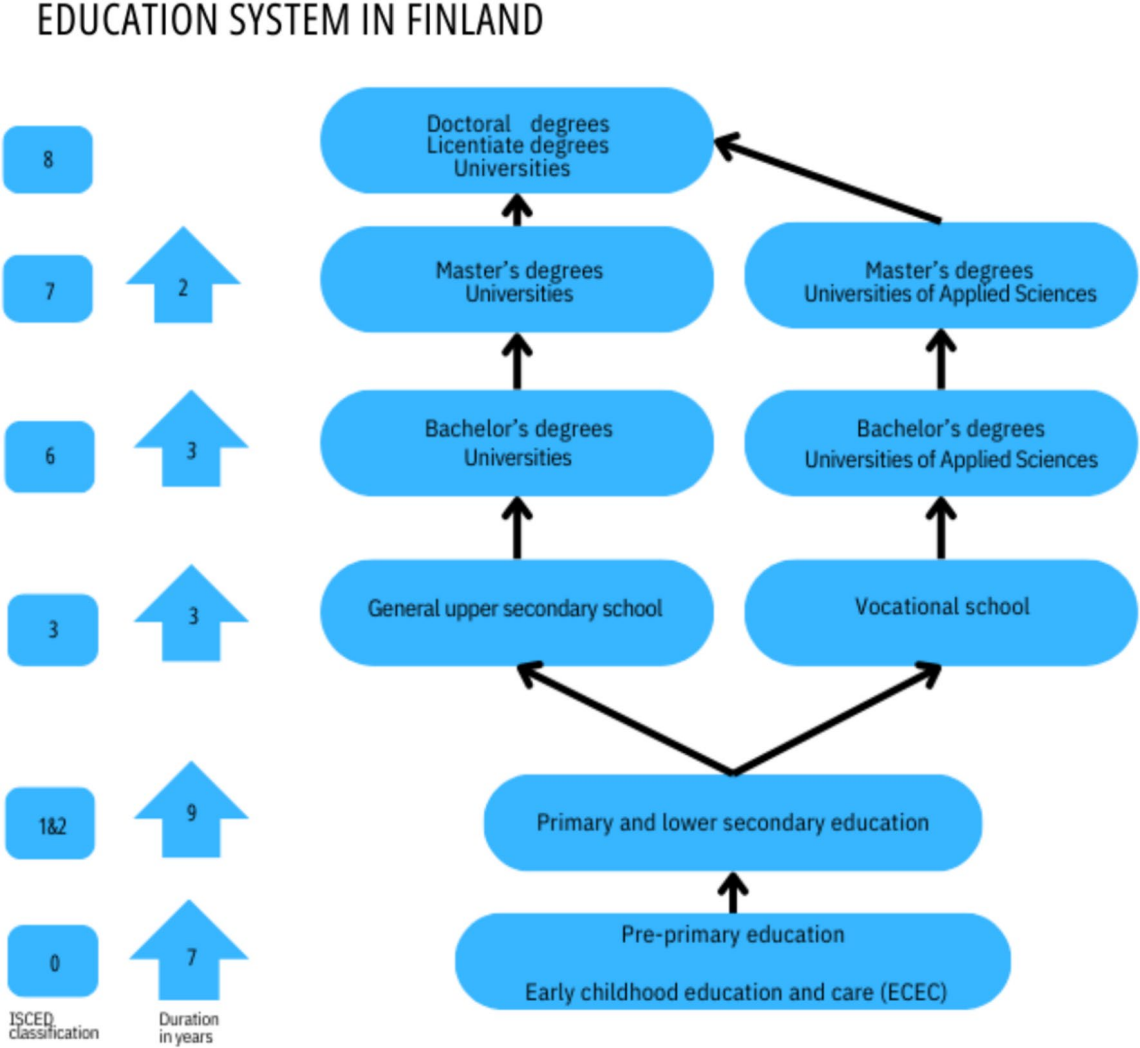
Different levels and duration of the finnish educational system from birth

Information on the highest educational attainment and educational institutions was gathered from Statistics Finland´s Degree/Qualification-data. The Finnish educational system consists of 5 main levels: early childhood education and care (ECEC), pre-primary education, primary and secondary education, upper secondary education, and tertiary education (Fig. 1). Primary and secondary education were compulsory in Finland during the follow-up [25]. Compulsory education usually starts at the age of 7 and continues to the end of secondary education, generally taking 9 years to complete [26]. It takes at least 25 years from birth to reach the highest tertiary education (licentiate/doctoral degree) and most men usually complete conscription (up to 1 year) at the age of 19 before attending tertiary education. For this reason, we excluded patients who were under 26 years of age at the end of the follow-up as these people would not have had time to complete the full length of the educational system. This excludes 0 to 4 year-old patients from our study population.

**Fig. 2:**
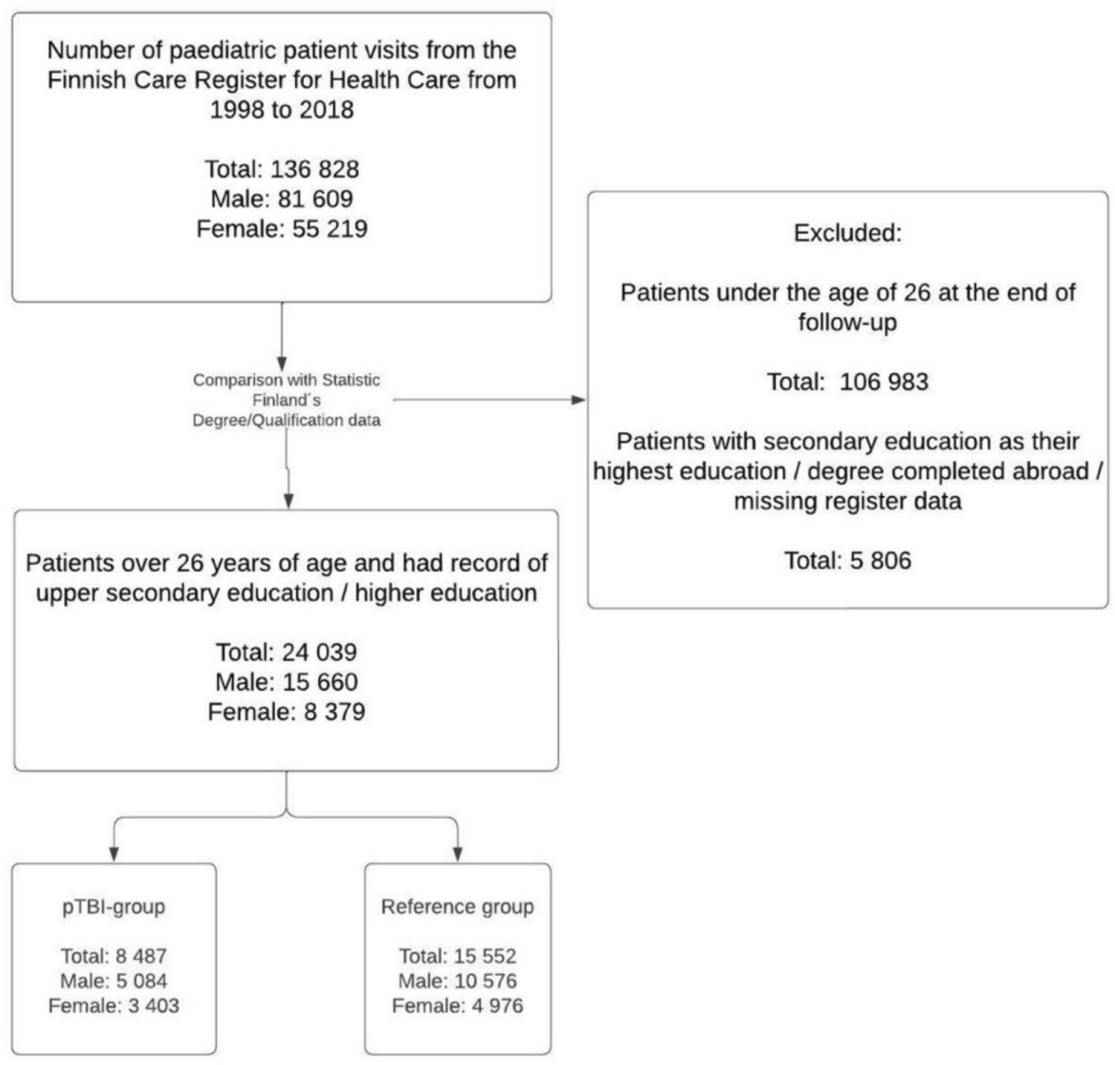
Flowchart of the formation of the study population

There were 5 806 patients in the Care Register for Health Care that were over 26 years of age but had no data on Degree/Qualification register. There are three possible reasons for the missing data: had secondary education as highest achieved education, completed upper secondary education and/or higher education abroad or had missing data in the register due to incomplete data linkage. As these registers use social security numbers to collect data from institutions, it is highly unlikely that the individuals would have missing data due to incomplete data linkage. However, it is not possible to point out the specific reason for the missing data as the Degree/Qualification register do not include information on lower educational levels such as secondary education. (Fig. 2).

We divided educational attainment into 3 main levels using the International Standard Classification of Education (ISCED): Upper secondary education (12 years of education), lower tertiary education (15 years of education), and higher tertiary education (+ 17 years of education). Upper secondary education was further divided into general upper secondary education and vocational training. Lower tertiary education was divided into university and polytechnic bachelor´s degrees. Higher tertiary education was divided into polytechnic and university master´s degrees as well as licentiate/doctoral degrees to represent the highest possible education. The data also contained information on all different subgroups of the educational institutions. All educational levels in Finland are free of charge. For a more detailed explanation of the educational levels as well as educational institutions, see Appendix 2.

### Statistical analyses

**Fig. 3:**
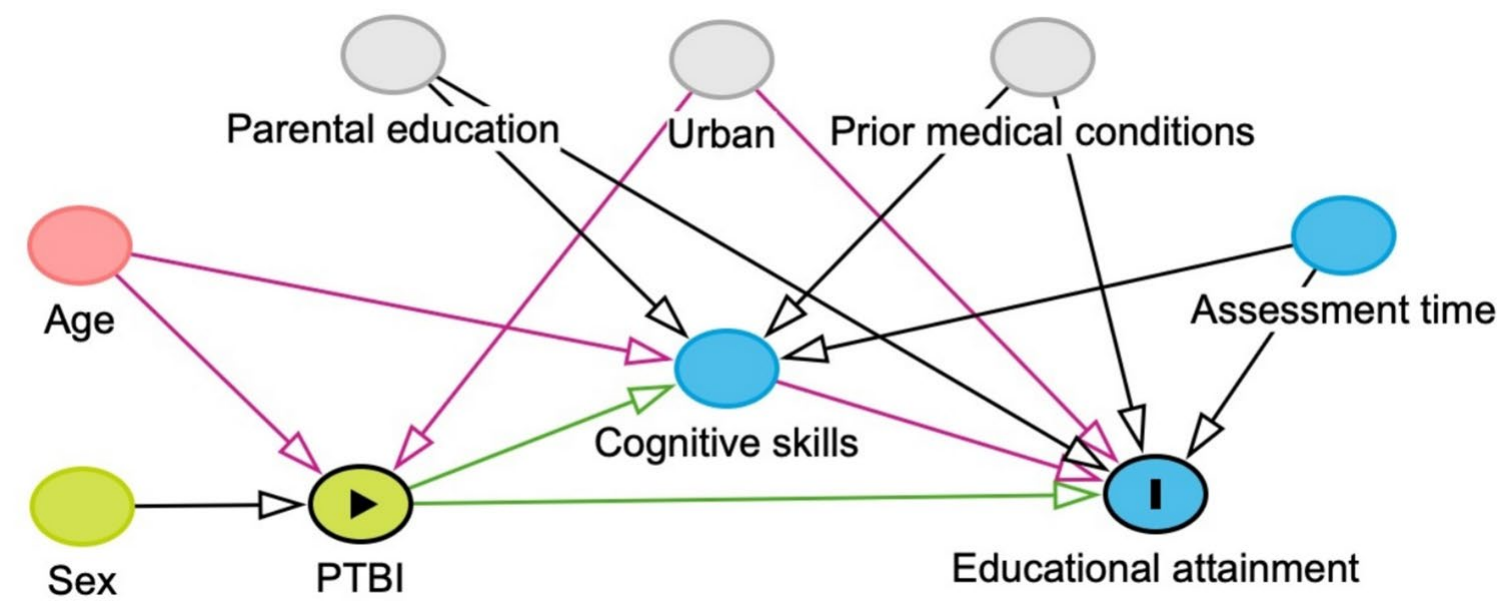
Directed acyclic graph of the impact of pTBI on educational attainment

Combined information from the Finnish Care Register for Health Care and the Statistics Finland´s Degree/Qualification-data were used to complete the study population using the patients’ unique identification numbers (Fig. 2). We compared the pTBI group and the reference group as well as concussions and specific intracranial injuries separately. We used logistic regression to calculate the odds ratio (OR) with 95% confidence intervals (CI) on the educational attainment between the pTBI group and the reference population. All analyses were adjusted by age, sex and year of injury diagnosis. Additionally, we conducted a sensitivity analysis and calculated the VanderWeele’s E-values for all ORs to describe the impact of unmeasured confounders [27, 28]. We compared upper secondary education to tertiary education, higher tertiary education to lower tertiary education, as well as general upper secondary education to vocational school (Fig. 3). We calculated percentages for all different types of educational institutions. All statistical analyses were performed using R version 4.0.5 (R Foundation for Statistical Computing, Vienna, Austria) [29].

## Results

**Fig. 4:**
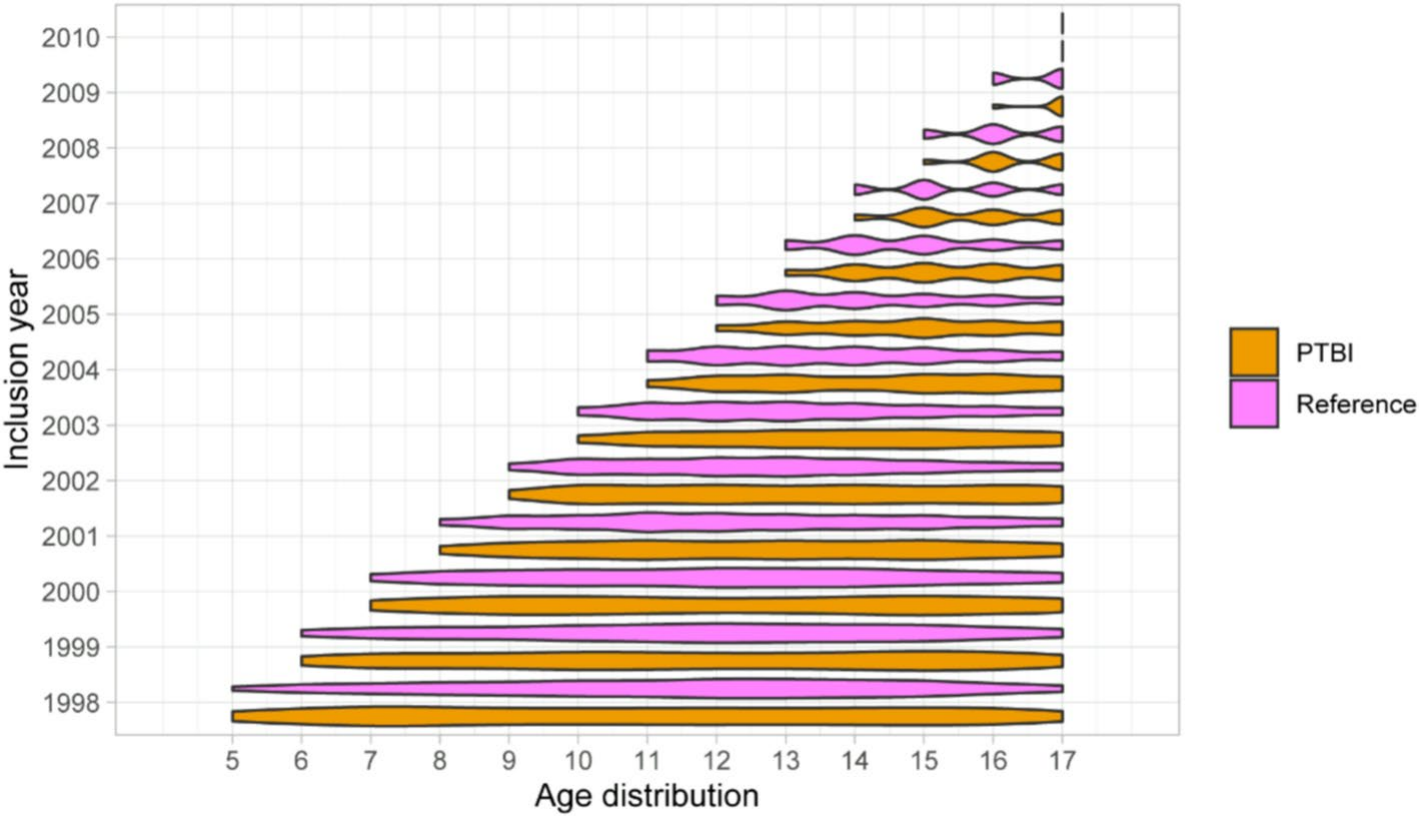
Age distribution by inclusion year between pTBI group and reference group

The research population comprised 8487 patients in the pTBI group and 15,552 patients in the reference group. In total, 7594 (89.5% of all pTBIs) patients in the pTBI group had a concussion and 892 (10.5% of all pTBIs) had a specific intracranial injury (Table 1). There were more men than women in both the pTBI (n = 5 084; 59.9%) and reference (n = 10 576; 68.0%) groups. Mean and median age at the time of injury was 13.2 and 13.0 years in the pTBI group and 12.9 and 13.0 years in the reference group. The age distribution was similar in both groups with slight over representation of adolescent population at the time of the initial injury (Fig. 4).

**Table 1:** Educational attainment compared between the pTBI group, the reference group, and those with concussions and specific intracranial injuries during the 21-years of follow-up

	pTBI group			Reference group
	Combined	Concussions	Specific intracranial injury	
Number of patients	8487 (100%)	7595	892	15,552
Upper secondary education (12 years of education)	5328 (62.8%)	4724 (62.2%)	604 (67.7%)	9155 (58.9%)
General upper secondary education	1164 (21.8%)	1061 (22.5%)	103 (17.1%)	2412 (26.3%)
Vocational training	4164 (78.2%)	3663 (77.5%)	501 (82.9%)	6743 (73.7%)
Lower tertiary education (15 years of education)	2164 (25.5%)	1972 (26.0%)	192 (21.5%)	4200 (27.0%)
Polytechnic Bachelor’s degree	1831 (84.6%)	1666 (84.5%)	165 (85.9%)	3546 (84.4%)
University Bachelor’s degree	333 (15.4%)	306 (15.5%)	27 (14.1%)	654 (15.6%)
Higher tertiary education (+ 17 years of education)	995 (11.7%)	899 (11.8%)	96 (10.8%)	2 197 (14.1%)
Polytechnic Master’s degree	31 (3.12%)	28 (3.11%)	3 (3.13%)	44 (2.00%)
University Master’s degree	934 (93.9%)	842 (93.7%)	92 (95.8%)	2098 (95.5%)
Licentiate/doctoral degree	30 (3.02%)	29 (3.00%)	1 (1.04%)	55 (2.50%)

The pTBI group had lower odds of attaining any tertiary education compared with the reference group (OR 0.80; CI 0.76, 0.85). The pTBI group also had lower odds of attaining higher tertiary education compared with the reference group (OR 0.77; CI 0.71, 0.83). Patients with specific intracranial injuries had lower odds of attaining any tertiary education compared to pTBI patients with concussions (OR 0.78; CI 0.67, 0.90). There was no notable difference in attaining higher tertiary education between pTBI patients with concussion compared to those with a specific intracranial injury (OR 0.85; CI 0.67, 1.05). Patients with concussion had lower odds of attaining any tertiary education compared to the reference group (OR 0.82; CI 0,78, 0.87). Concussion patients also had lower odds of attaining higher tertiary education compared to the reference group (OR 0.78; CI 0.72, 0.85). (Table 1).

The most common educational institutions for highest achieved education were vocational schools, universities of applied sciences, and universities in both groups (Table 2). A total of 5328 (62.8%) patients in the pTBI group and 9155 (58.9%) patients in the reference group had upper secondary education as their highest education during our 21-year follow-up. When comparing the educational institutions, the pTBI group had lower odds of attaining general upper secondary education than vocational training (OR 0.78; CI 0.72, 0.85). Pediatric TBI patients with a specific intracranial injury had lower odds of attaining general upper secondary education than attending vocational training compared to pTBI patients with concussion (OR 0.78; CI 0.62, 0.97). Concussion patients had lower odds of attaining general upper secondary education than reference group patients (OR 0.80; CI 0.74, 0.87). (Table 1).

**Table 2:** Educational institutions attended by the pTBI and reference groups

	pTBI group	Reference group
Number of patients	8487	15,552
General vocational schools	3647 (43.0%)	5930 (38.1%)
Universities of applied sciences	1852 (21.8%)	3575 (23.0%)
Universities	1269 (15.0%)	2756 (17.7%)
General upper secondary school	1088 (12.8%)	2235 (14.4%)
Unspecified	624 (7.36%)	1056 (6.79%)

Patients in unspecified institutions have a degree in vocational institutions but the specific institution (fire department, police training, general vocational school, physical education, music schools) could not be verified

VanderWeele´s E-values varied between 1.74–1.92, CI 1.21–1.70. All E-values are presented with more detail in Appendix 1.

## Discussion

Children/adolescents with a history of pTBI had lower educational attainment than our reference population with orthopedic injuries. People in the pTBI group did not move from upper secondary education to tertiary education as often as the reference group. People in the pTBI group also did not move from lower tertiary education to higher tertiary education as often as the reference group. When evaluating people within the pTBI group, specific intracranial injuries were associated with a lower educational attainment than concussions. In contrast, concussion patients had lower educational attainment than reference group.

Our comparisons were done using a unique reference group with injury background. Our hypothesis was that mimicking risk taking behavior we could see more reliable results for the impact of TBI to educational attainment. According to Statistics Finland, 50.1% of 26-year-old general population had a degree in upper secondary education in 2018 [30]. Total of 32.1% have a degree in lower tertiary education and 17.8% has degree in higher tertiary education [30]. As we can see in these statistics, the general population has notably higher proportion of tertiary education degrees compared to pTBI and reference group that supports our hypothesis.

Previous literature on this topic is scarce, but our results regarding the association of pTBI with lower educational attainment are consistent with earlier reports. A previous nationwide Swedish cohort study reported that adolescents with a history of pTBI did not attain secondary education to the level of the general population [19]. Swedish and Finnish education systems are similarly constructed and both are free of charge, making the data comparable [31]. Our study had more educational levels to compare as well as a more comparable reference population comprising people with an injury background.

An extensive Scottish cohort study showed a large scale of adverse educational outcomes that were more common in those with a TBI background [17]. Children/adolescents with a history of TBI needed more special education, were absent more often, had lower education attainment, and were excluded from school more than the general population [17]. Several studies implicated a negative longer-term impact on memory function, cognitive flexibility, and mental health problems, with the impact increasing as the severity of the TBI increases [15, 16, 18, 32]. Our study findings enhance these findings showing that the possible far-reaching impact on educational attainment could extend from the upper secondary level of education to the tertiary level.

Especially the incidence of mild pTBI has been on the rise in Finland during the study period [5]. In our data, pediatric patients with concussions had considerably lower educational attainment at all higher levels than reference population. Concussions are the mildest diagnose code in this injury category and implicate a mild TBI with no severe symptoms/imagining findings. Dipnall et all also found that minor TBI had negative impact on educational attainment which was not found on wrist/ankle injury patients [21] The difference was considerable more clearer in our data. Notably, a negative impact on educational attainment was also seen in pTBI patients with specific intracranial injuries compared to those with concussion. This could implicate a dose–response relationship, i.e., the impact is greater with more serious injuries.

Upper secondary education starts around 16 years of age in Finland. At this age, Finnish students make a big decision in their lives to go either on a more academic route of general upper secondary school or vocational education and training. In our study, the pTBI group considerably more often chose vocational training compared with the reference population. This difference was also seen with possibly more serious, specific, intracranial injuries compared with concussions. Our study groups’ mean ages were well under 16, suggesting that most these injuries occurred under the age of 16 and possibly affected the decision regarding these further educational routes.

This study has multiple strengths. The Finnish Care Register for Health Care has excellent coverage and quality [33]. The ICD-10 classification remained the same throughout the study period and the consistency of marking these codes to registers is excellent. The Finnish educational system is well constructed, free of charge, and underwent no major alterations during our study period. We had extensive and broad data that comprised all higher educational levels in Finland and allowed reliable comparisons. Even though socioeconomic status influences access to education, free education that is financially available to everyone makes it more equally achievable. A weakness of our study is the lack of specific information on pTBI severity as well as reliable information on people who missed upper secondary education. Our analyses were only adjusted for age, sex and year of the injury, lacking information on other potential confounders such as parental education, neighborhood/urban or other prior/childhood mental or physical morbidity (Fig. 3). In sensitivity analyses, we studied the impact of potential unmeasured confounders in form of the E-values (Appendix 1). The calculated E-values indicate that the impact of unmeasured confounders would have to be highly notable in order to explain away our effect estimate.

## Conclusion

People with pTBI had lower educational attainment at all higher educational levels than the reference population with orthopedic injuries. The educational attainment was lower regardless of the injury severity. People with specific intracranial injuries had lower attainment in tertiary education than people with concussions. For future monitoring of pTBI patients, it is important to pay attention to the possible long-term negative cognitive impact that can lead to lower educational attainment.

## Appendix 1: Tables containing ICD-10 diagnose codes used in the study as well as all E-values calculated in sensitivity analyses

See Tables

**Table 3:** Diagnose codes used to identify pTBI (ICD-10 diagnostic code system)

Diagnose code	Definition
S06.0	Concussion
S06.1	Traumatic cerebral edema
S06.2	Diffuse traumatic brain injury
S06.3	Focal traumatic brain injury
S06.4	Epidural hemorrhage
S06.5	Traumatic subdural hemorrhage
S06.6	Traumatic subarachnoid hemorrhage
S06.7	Intracranial injury and prolonged concussion
S06.8	Other intracranial injury
S06.9	Unspecified intracranial injury

3,

**Table 4:** Diagnose codes used to identify the reference group of wrist and ankle fractures

Diagnose code	Definition
S52.0	Fracture of upper end of ulna
S52.1	Fracture of upper end of radius
S52.2	Fracture of shaft of ulna
S52.3	Fracture of shaft of radius
S52.4	Fracture of shaft of radius and ulna
S52.5	Fracture of lower end of radius
S52.6	Fracture of lower end of radius and ulna
S52.7	Multiple fractures of ulna
S52.8	Fracture of other part of ulna
S52.9	Unspecified fracture of forearm
S82.0	Fracture of patella
S82.1	Fracture of upper end of tibia
S82.2	Fracture of shaft of tibia
S82.3	Fracture of lower end of tibia
S82.4	Fracture of shaft of fibula
S82.5	Fracture of medial malleolus
S82.6	Fracture of lateral malleolus
S82.7	Multiple fractures of tibia or fibula
S82.8	Other fractures of lower leg
S82.9	Unspecified fracture of lower leg

4 and

**Table 5:** Sensitivity analysis assessing the influence of unmeasured confounders using VanderWeele’s E-values

Comparison between pTBI and reference group	Odds ratio	E-value
Attaining any tertiary education	0.80, CI 0.76, 0.85	1.81, CI 1.63
Attaining higher tertiary education	0.77, CI 0.71, 0.83	1.92, CI 1.70
Attaining upper secondary education	0.78, CI 0.72, 0.85	1.88, CI 1.63
*Comparison between concussions and reference*		
Attaining any tertiary education	0.82, CI 0,78, 0.87	1.74, CI 1.56
Attaining higher tertiary education	0.78, CI 0.72, 0.85	1.88, CI 1.63
Attaining upper secondary education	0.80, CI 0.74, 0.87	1.81, CI 1.56
*Comparison between concussions and specific intracranial injuries*		
Attaining any tertiary education	0.78, CI 0.67, 0.90	1.88, CI 1.46
Attaining higher tertiary education	0.85, CI 0.67, 1.05	1.63, CI 1.00
Attaining upper secondary education	0.78, CI 0.62, 0.97	1.88, CI 1.21

^5^.

## Appendix 2: The finnish educational system

The educational system starts with early childhood education and care before the age of six (non-compulsory). Then children can attain pre-primary education at the age of six before the start of compulsory education. Compulsory education consists of nine-years of primary and secondary education (comprehensive school). After nine years of comprehensive school children decide on going one of the two possible main lines in upper secondary education that are either general upper secondary education or vocational education and training. General upper secondary education does not qualify students for any occupation. Vocational qualifications give the students basic skills required for the studied occupation (e.g. car mechanic, electrician, practical nurse). There are also special types of vocational institutes such as physical education, fire, police, and security training institutions and music schools that have their own special application programs. In this study, we divided people to all these special institutions (see. Table 2).

Higher education (tertiary education) is provided by universities and universities of applied sciences. To get selected to a university program, you need either good certificate of matriculation from general upper secondary school or good success on entrance exams. For universities of applied sciences, you need to have general upper secondary certificate or vocational training certificate. University degrees usually consist of 3-year bachelor´s degree and 2-year master´s degree. In universities of applied sciences, the degrees usually only consist of 3-year bachelor´s degrees with an option to study more in university of applied sciences to get master´s degree or apply to university´s master´s program in selected fields. Programs that lead to licentiate and doctoral degrees are only available in universities. For more detailed information on educational system in Finland, please visit Finland´s ministry of education and culture: https://okm.fi/en/education-system.

For our studies main analyses, we divided people in to three levels of above-mentioned educational levels: upper secondary education, lower tertiary education and higher tertiary education. Lower tertiary education contains all university and university of applied sciences bachelor´s degrees. Higher tertiary education contains university and university of applied sciences master´s degrees as well as higher university degrees of licentiate degrees and doctoral degrees.
